# Supplementary material for: Effects of self-isolation and quarantine on loot box spending and excessive gaming—results of a natural experiment
Source: PeerJ. 2021 Feb 3;9:e10705. doi: 10.7717/peerj.10705 (PMC7866886; doi:10.7717/peerj.10705)
Supplement: Supplemental Information 1 [file peerj-09-10705-s001.docx]

**Online Supplementary Materials**

**Methods**

**Measures**

We pre-registered comparisons between two separate dichotomised groups – those who were self-isolating (limiting contact with other people) or quarantined (under mandated self-isolation) and those that were not, and those who were under lockdown orders (in cities where movement and gathering restrictions were in place) and those who were not. Unexpectedly, very few participants were not in lockdown (*n* = 69, ~6% of our total data; 1,075 participants were in lockdown). Thus, although we choose to provide the results of these pre-registered comparisons, we emphasise that these estimates for non-lockdown participants are unreliable due to the very small sample size, and must be interpreted with caution. The almost ubiquitous presence of lockdown orders in our data makes it difficult to examine the effects of lockdown on our dependent measures. Results for pre-registered comparisons between participants in lockdown and participants not in lockdown are presented in this supplementary document, though we urge caution when interpreting these findings due to the small sample size and the risk of over-interpreting (probably) unreliable estimates.

**Results**

Very few participants were not in lockdown (*n* = 69). The results which follow may be unreliable due to the very small sample size. We urge caution when interpreting these results. We found no evidence that psychological distress was higher for those in lockdown, *t*(1,142) = 0.63, *p* = .531, *d* = .08. Lockdown was associated with increased loot box spending with participants in lockdown spending significantly more on loot boxes (*M* = $6.20 USD, *SD* = $20.25 USD) than participants not in lockdown (*M* = $2.77 USD, *SD*= $10.65 USD), *t*(1,133) = 2.41, *p* = .016, *d* = 0.30. However, lockdown was not associated with increased excessive gaming, or risky loot box use. The relationships between contamination concern, loot box spending, IGDC and RLI, split by lockdown status is reported in Table S1.

Table S1. Associations between Contamination Concern and Problematic Gaming Scales according to Lockdown status for All Respondents (first column), Participants Not in Lockdown (second column), and In Lockdown (third column). Z-tests (column four) reported for the difference between participants in Lockdown and not in Lockdown. Correlations reported in Spearman’s Rho.

|  | **Contamination Concern** | | | |
| --- | --- | --- | --- | --- |
|  | **All Respondents** | **Not in Lockdown** | **In Lockdown** | **Z-test for difference** |
| **Loot Box Spend** | .163** | .282* | .155** | Z = 1.053,  *p* = .292 |
| **RLI** | .247** | .492** | .232** | Z = 2.384,  *p* = .017* |
| **IGD** | .263** | .491** | .248** | Z = 2.240,  *p* = .025* |

**p* < .05, ***p* < .001

Problem gambling symptoms were associated with higher loot box spending, *r_s_* = .279, *p* < .001, which was similar for those in lockdown, *r_s_* = .275, *p* < .001, than not, *r_s_* = .321, *p* = .007, difference between correlation strengths, Z = 0.398, *p* = .691. We found no evidence that lockdown status altered the relationship between loot box spending and problem gambling group status.
